# Supplementary material for: Knowledge, attitudes and practices towards malaria in a peri-urban Cameroonian village: Persistent silent transmission despite satisfactory awareness
Source: PLoS One. 2026 May 27;21(5):e0348617. doi: 10.1371/journal.pone.0348617 (PMC13215510; doi:10.1371/journal.pone.0348617)
Supplement: Attachment — Submitted filename: A REVIEW REPORT by EAA.docx [file pone.0348617.s005.docx]

A REVIEW REPORT ON KNOWLEDGE, ATTITUDES AND PRACTICES TOWARDS MALARIA IN CAMEROONIAN VILLAGE: LOW AWARENESS AND SILENT TRANSMISSION AMONG YOUTH BY DR. EMMANUEL AKPAN

Abstract:

The abstract is generally clear and well-written, providing a good overview of the study’s objective, methods, results, and conclusion. However, the overall malaria prevalence of 24.9% is smaller compared to that of a particular age group (5–18 years), which is 34%.

Way Forward

Recalculate the overall prevalence. Taking age, for instance, the overall prevalence should be the sum of the prevalence for each age group.

Introduction/Background:

The introduction is well-written, capturing a strong statement of the problem and outlining clear objectives of the study.

Results:

There are inconsistencies in the results. There are cases of fractional parts of percentage values being mixed up, and commas used in place of points across all the Tables. There is also a mix-up in the use of sample size and number of households.

Way Forward

1. Authors are required to correct all as appropriate. For example, in Table 1, the row for sex, Female 31(24,9%), is corrected to 31(24.9%). All analyses should be done with respect to sample size, not the number of households.
2. The focus is on salient transmission among youth as reflected in the title, but participants of all ages are included in the study. The authors may need to make the necessary adjustments.
3. In most Tables, especially Table 6, their frequency counts are not consistent and are not equal to the sample size.
4. Lines 207-208, the mean values presented should not be further divided by different scores so as to tally with the mean values in Table 2.
5. The rationales for the adoption of different statistical tools should be provided.

Limitations:

Discuss the limitations, including potential sampling biases and issues related to the generalizability to larger populations.
