# Supplementary material for: Knowledge, attitudes and practices towards malaria in a peri-urban Cameroonian village: Persistent silent transmission despite satisfactory awareness
Source: PLoS One. 2026 May 27;21(5):e0348617. doi: 10.1371/journal.pone.0348617 (PMC13215510; doi:10.1371/journal.pone.0348617)
Supplement: Attachment — Submitted filename: Response to Reviewers.docx [file pone.0348617.s006.docx]

Date: December 29, 2025
The Editor-in-Chief
PLoS ONE
PLOS
[1160](tel:1160) Battery Street
Ketchum Suite [500](tel:500)
San Francisco, CA [94111](tel:94111)

**Subject**: Point-by-point response to the reviewed manuscript PONE-D-25-49635

Dear Academic Editor and PLOS ONE Editorial Team,

Thank you for your thorough review and constructive feedback on our manuscript titled "**Knowledge, Attitudes, and Practices Towards Malaria in a Cameroonian Village: Low Awareness and Silent Transmission Among Youth".** We have carefully addressed each comment below, with detailed revisions made to the manuscript. All changes are highlighted in the attached Track Changes version (Revised [Manuscript with Track Changes.docx](http://Manuscript_Track_Changes_PONE-D-25-9635.docx)). A clean revised manuscript named “Manuscript” is also uploaded.

**a.Editor comment**: Pease ensure that your manuscript meets PLOS ONE’s style requirements, including those for file naming.

**Response**: We thing that our manuscript now meets PONE ONE’s style requirements and we have name files appropriately.

**b. concerning the figure of the study site in our manuscript**

**Editor comment**: a copyright should be available

**Response**: The map was entirely created by the corresponding author using ArcMap software (Esri software) during the preparation of this study. All data sources used for the map (e.g., administrative boundaries, roads, rivers, geographical coordinate points or village location) are public domain or open-access geaospacial data from freely available repositories, including 1- Cameroon administrative boundaries and topographic data from the Humanitarian Data Exchange (HDX) – <https://data.humdata.org> (public domain under CCO or similar open license). No copyright or third-party licensed material (e.g. commercial imagery, restricted datasets) was used. The map layout, labels, legend, and all design elements were original work produced by the author.

Therefor, we hold full copyright as the creator of the figure. We hereby grant PLOS ONE a non-exclusive license to publish and reproduce the map as part of the article under your standard open-access license (CC BY 4.0), with appropriate credit to the author.

**c. Editor comment**: We not that your Data Availability Statement is currently as follows: All relevant data are within the manuscript and it’s supporting information files. {…} Author must share their “minimal dataset” for their submission required to replicate the results of your study.

**Response**: We shared the K.A.P datasets and the parasitological dataset in “other files”

**1.Complete revision due to numerous spelling and grammatical errors**

**Reviewer 1 comment:** The text requires complete revision due to numerous spelling and grammatical errors.

**Response**: The text was entirely checked and spelling and grammatical errors were corrected.
**2. Abstract – Overall prevalence vs age group prevalence
Reviewer 1 comment**: The overall malaria prevalence of [24.9](tel:24.9)% is smaller compared to that of a particular age group (5-18 years), which is 34%. Recalculate the overall prevalence. Taking age, for instance, the overall prevalence should be the sum of the prevalence for each age group.
**Response**: We thank the reviewer for this observation. The overall prevalence of [24.9](tel:24.9)% is correctly calculated as the proportion of positive slides among the total sample tested (62 positives / [249](tel:249) participants). The prevalence in the 5-18 years group was recalculated (13.25%). We have revised this in the revised manuscript (Tableau 1, lines 205-206).
**3. Results - Inconsistencies in percentage formatting, commas vs points, and sample size vs household number**
**Reviewer comment**: There are cases of fractional parts of percentage values being mixed up, and commas used in place of points across all the Tables. There is also a mix-up in the use of sample size and number of households.
**Response**:
We apologize for these formatting inconsistencies and have corrected them throughout the manuscript in all tables.
All decimal separators are now points (e.g., [24.9](tel:24.9)% instead of 24,9%).
Percentages are consistently rounded conveniently (e.g., [24.9](tel:24.9)%, [34.0](tel:34.0)%).
All analyses and frequencies are now explicitly based on the correct sample size (N=[249](tel:249) for parasitological data; N=[104](tel:104) for sociodemographic and housing variables with complete data). References to "number of households" have been corrected to "number of individual" in a household in Table 11.
These changes are visible in the Track Changes version (line 307-308).
**4. Title and focus - Youth vs all ages included
Reviewer 1 & 2 comment**: The focus is on salient transmission among youth as reflected in the title, but participants of all ages are included in the study. The authors may need to make the necessary adjustments.
**Response**:
We appreciate this feedback and agree that the title should accurately reflect the study's scope. The study included participants of all ages for a comprehensive population assessment. To align the title with the content and avoid misleading emphasis on youth alone, we have revised the title to:
**" Knowledge, attitudes and practices towards malaria in a peri-urban Cameroonian village: persistent silent transmission despite satisfactory awareness**"
This better reflects the general population focus while retaining the key finding. The revised title appears in the manuscript header and abstract (line 1). We have also clarified throughout the text that the study population includes all ages, with age-stratified prevalence highlighted as a subgroup finding.
**5. Tables - Frequency counts inconsistent and not equal to sample size (e.g., Table 6)**
**Reviewer comment 2 & 1**: In most Tables, especially Table 6, their frequency counts are not consistent and are not equal to the sample size.
**Response**:
We have carefully rechecked and corrected all tables for consistency. Frequency counts now sum exactly to the stated sample size (N=[249](tel:249) for parasitological tables; N=[104](tel:104) for sociodemographic/housing tables). Missing or incomplete data are now explicitly noted as "NA". For example, Table 6 has been revised to ensure totals match , except for the variables which have the “*” asterisk indicating a multiple choices or responses. All changes are tracked (Tables 1–10 revised).
**6. Lines** [**207**](tel:207)**-**[**208**](tel:208) **– Mean values and score division
Reviewer comment 2**: Lines [207-208](tel:207-208), the mean values presented should not be further divided by different scores so as to tally with the mean values in Table 2.
**Response**:
This was an editorial error. We have removed any inappropriate division of means by sub-scores and ensured consistency with Table 2. The revised text (lines 211–217) reads:
"Knowledge scores averaged [5.11](tel:5.11) (SD [1.35](tel:1.35)), attitudes [6.67](tel:6.67) (SD [2.03](tel:2.03)), and practices [3.54](tel:3.54) (SD [0.65](tel:0.65)), as detailed in Table 2."
**7. Rationales for statistical tools**
**Reviewer comment** : The rationales for the adoption of different statistical tools should be provided.
**Response**: We have added a dedicated paragraph in the Methods section (Statistical analysis subsection, page 8, lines 158-196) explaining the choice of tests:
"Due to the non-normal distribution of KAP scores (Shapiro-Wilk test), non-parametric tests were used: Mann-Whitney U for two-group comparisons (e.g., sex), Kruskal-Wallis H for multiple groups (e.g., age, occupation, education), and Spearman's rho for correlations (e.g., household size). For binary parasitological prevalence, Pearson's chi-square or Fisher's exact test was applied when appropriate, with univariate logistic regression for crude odds ratios (COR) and 95% CI. These choices preserved data integrity and avoided assumptions violated by parametric tests."
This rationale is explicit and justified.
**8. Discussion wrote according reviewer 1’s plan, Strengths, Limitations and implication**
**Reviewer 1 & 2 comments**: **R1**-Discuss the limitations, including potential sampling biases and issues related to the generalizability to larger populations. **R2**- The discussion is rather disjointed and seems to have strayed from the initial objectives of the study.
**Response**: The discussion was rewritten accordingly to the proposed plan by Reviewer 1.
A new subsections "Strengths and Limitations", “Implication for clinicians and policymakers” and “future research” were been added to the Discussion (pages 20, lines 320-378):
"Strengths include the community-based integration of parasitological and KAP data in a peri-urban setting. Limitations encompass the modest sample size (N=[104](tel:104) for sociodemographics), limiting power for subtle associations. Multivariate modeling was not performed due to sparse positives and non-significant univariates. Self-reported KAP may introduce social desirability bias, though triangulation with prevalence mitigates this. Findings are specific to Emana village and may not generalize to larger or more rural populations due to peri-urban context and potential sampling biases (convenience sampling)." This addresses generalizability and biases directly.

**9.The references should be reviewed**

**Reviewer 1**: the references should be reviewed, particularly numbers 1,3,10,13,17,21,23 and 28.

**Response**: Thank you for pointed this. An error occurs when sending the first manuscript and some references were lost. They were now been checked entirely and all been cited bot in the text and in bibliography.

**Reviewer 1 comment:** Why Emana was chose? And why wait four years to publish the results?

**Response**: Here we make a mistake, the study was realized in 2023 not in 2022, we are really sorry for that. Emana was chosen because it is a sparsely populated and diverse village (with children, young people and adults), which facilitates implementation. It is also a locality where malaria is prevalent and widespread, with high density of vectors. We wanted to understand why malaria is prevalent there and what factors, besides the high vector density, could explain it.

This study was delayed in its publication due to personal reasons that prevented the corresponding author from publishing it.

**10. About the term ‘hemophilic”**

**Reviewer 1 comment**: what do you mean by hemophilic diseases ? how could participants with limited medical knowledge tell the difference between this term and other medical conditions?

**Response**: Thank you for pointing that out. We were referring to the disease “Hemophilia” here; it was a typographical error. Therefore, people who might have this disease should not have been included in the study.
**11. About some others remarks by reviewer 1**

**Response**: in tab 8, all values were now indicated in $(page 15, line 285-287), we prefer keep bot the p-value and the statistic test values rho, Mann-Whitney Kruskal-Wallis because they are together more informative in the sound of our fundings in the relevant tables 5,7,9,10,11. Abbreviations were checked (e.g., LLINs was employed everywhere in the text instead of “ITNs”, page 3, line 54), corrected and are now consistent in the manuscript. The term “March” was corrected (page 5, line 120).

We believe these revisions fully address all concerns raised. Thank you again for your valuable feedback, which has significantly improved the manuscript. Please let us know if any further clarification is needed.
Sincerely,
Steve Joko Tamoufé
OCEAC/University of Dschang, email
On behalf of all authors
