# Supplementary material for: Knowledge, attitudes and practices towards malaria in a peri-urban Cameroonian village: Persistent silent transmission despite satisfactory awareness
Source: PLoS One. 2026 May 27;21(5):e0348617. doi: 10.1371/journal.pone.0348617 (PMC13215510; doi:10.1371/journal.pone.0348617)
Supplement: Attachment — Submitted filename: Review Recommendation.docx [file pone.0348617.s007.docx]

A RECOMMENDATION ON KNOWLEDGE, ATTITUDES AND PRACTICES TOWARDS MALARIA IN PERI-URBAN CAMEROONIAN VILLAGE: PERSISTENT SILENT TRANSMISSION DESPITE SATISFACTORY AWARENESS BY DR. EMMANUEL AKPAN

### Comments

### The authors have responded adequately to all my review comments, and the manuscript can be accepted for publication.

Abstract:

The abstract is generally clear and well-written, providing a good overview of the study’s objective, methods, results, and conclusion. However, the overall malaria prevalence of 24.9% is smaller compared to that of a particular age group (5–18 years), which is 34%.

Way Forward

Recalculate the overall prevalence. Taking age, for instance, the overall prevalence should be the sum of the prevalence for each age group.

**Response**

## We thank the reviewer for this observation. The overall prevalence of 24.9% is correctly calculated as the proportion of positive slides among the total sample tested (62 positives / 249 participants). The prevalence in the 5-18 years group was recalculated (13.25%). We have revised this in the revised manuscript (Tableau 1, lines 205-206).

## The malaria prevalence for the 5-18-year age group has been recalculated from 34% to 13.25%, which is now correct as required. I am satisfied with the response provided.

Introduction/Background:

The introduction is well-written, capturing a strong statement of the problem and outlining clear objectives of the study.

Results:

There are inconsistencies in the results. There are cases of fractional parts of percentage values being mixed up, and commas used in place of points across all the Tables. There is also a mix-up in the use of sample size and number of households.

Way Forward

1. Authors are required to correct all as appropriate. For example, in Table 1, the row for sex, Female 31(24,9%), is corrected to 31(24.9%). All analyses should be done with respect to sample size, not the number of households.

**Response**

We apologize for these formatting inconsistencies and have corrected them throughout the manuscript in all tables. All decimal separators are now points (e.g., 24.9% instead of 24,9%). Percentages are consistently rounded conveniently (e.g., 24.9%, 34.0%). All analyses and frequencies are now explicitly based on the correct sample size (N=249 for parasitological data; N=104 for sociodemographic and housing variables with complete data). References to "number of households" have been corrected to "number of individual" in a household in Table 11. These changes are visible in the Track Changes version (line 307-308).

### All required corrections are effected with a great level of satisfaction

2. The focus is on salient transmission among youth as reflected in the title, but participants of all ages are included in the study. The authors may need to make the necessary adjustments.

**Response**

appreciate this feedback and agree that the title should accurately reflect the study's scope. The study included participants of all ages for a comprehensive population assessment. To align the title with the content and avoid misleading emphasis on youth alone, we have revised the title to: " Knowledge, attitudes and practices towards malaria in a peri-urban Cameroonian village: persistent silent transmission despite satisfactory awareness" This better reflects the general population focus while retaining the key finding. The revised title appears in the manuscript header and abstract (line 1). We have also clarified throughout the text that the study population includes all ages, with age-stratified prevalence highlighted as a subgroup finding.

### The title has been rephrased to capture the scope of the study. I totally agree with the amended title.

3. In most Tables, especially Table 6, their frequency counts are not consistent and are not equal to the sample size.

**Response**

We have carefully rechecked and corrected all tables for consistency. Frequency counts now sum exactly to the stated sample size (N=249 for parasitological tables; N=104 for sociodemographic/housing tables). Missing or incomplete data are now explicitly noted as "NA". For example, Table 6 has been revised to ensure totals match , except for the variables which have the “*” asterisk indicating a multiple choices or responses. All changes are tracked (Tables 1–10 revised).

### I am satisfied with the corrections made.

4. Lines 207-208, the mean values presented should not be further divided by different scores so as to tally with the mean values in Table 2.

**Response**

This was an editorial error. We have removed any inappropriate division of means by sub scores and ensured consistency with Table 2. The revised text (lines 211–217) reads: "Knowledge scores averaged 5.11 (SD 1.35), attitudes 6.67 (SD 2.03), and practices 3.54 (SD 0.65), as detailed in Table 2."

### Corrected as required with a good satisfaction.

5. The rationales for the adoption of different statistical tools should be provided.

**Response**

We have added a dedicated paragraph in the Methods section (Statistical analysis subsection, page 8, lines 158-196) explaining the choice of tests: "Due to the non-normal distribution of KAP scores (Shapiro-Wilk test), non-parametric tests were used: Mann-Whitney U for two-group comparisons (e.g., sex), Kruskal-Wallis H for multiple groups (e.g., age, occupation, education), and Spearman's rho for correlations (e.g., household size). For binary parasitological prevalence, Pearson's chi-square or Fisher's exact test was applied when appropriate, with univariate logistic regression for crude odds ratios (COR) and 95% CI. These choices preserved data integrity and avoided assumptions violated by parametric tests." This rationale is explicit and justified.

### I am satisfied with the justification provided.

Limitations:

Discuss the limitations, including potential sampling biases and issues related to the generalizability to larger populations.

**Response**

The discussion was rewritten accordingly to the proposed plan by Reviewer 1. A new subsections "Strengths and Limitations", “Implication for clinicians and policymakers” and “future research” were been added to the Discussion (pages 20, lines 320-378): "Strengths include the community-based integration of parasitological and KAP data in a peri-urban setting. Limitations encompass the modest sample size (N=104 for sociodemographics), limiting power for subtle associations. Multivariate modeling was not performed due to sparse positives and non-significant univariates. Self-reported KAP may introduce social desirability bias, though triangulation with prevalence mitigates this. Findings are specific to Emana village and may not generalize to larger or more rural populations due to peri-urban context and potential sampling biases (convenience sampling)." This addresses generalizability and biases directly.

### The discussion of limitations has been provided and is satisfactory.
